# Evaluation of Terpene Decomposition in Kaffir Lime Juice during Storage Using Gas Chromatography–Mass Spectrometry and Proton Transfer Reaction–Mass Spectrometry

**DOI:** 10.3390/molecules29133241

**Published:** 2024-07-08

**Authors:** Martyna Lubinska-Szczygeł, Żaneta Polkowska, Blanka Tobolkova, Tomasz Majchrzak, Martin Polovka, Parichart Promchote, Shela Gorinstein

**Affiliations:** 1 Department of Analytical Chemistry, Faculty of Chemistry, Gdańsk University of Technology, 80-233 Gdańsk, Poland; tomasz.majchrzak@pg.edu.pl; 2 Department of Chemistry and Food Analysis, National Agricultural and Food Centre-Food Research Institute, 824 75 Bratislava, Slovakia; blanka.tobolkova@nppc.sk (B.T.); martin.polovka@nppc.sk (M.P.); 3 Department of Agronomy, Faculty of Agriculture, Kasetsart University, Chatuchak, Bangkok 10900, Thailand; parichart.pr@ku.th; 4 Institute for Drug Research, School of Pharmacy, Faculty of Medicine, The Hebrew University of Jerusalem, Jerusalem 9112001, Israel; shela.gorin@mail.huji.ac.il

**Keywords:** kaffir lime, terpenes, decomposition, proton transfer reaction mass spectrometry, gas chromatography

## Abstract

Kaffir lime juice, often treated as production waste, can be a good source of terpenes. These compounds undergo various decomposition processes under the influence of external factors, especially during transportation and storage. In this paper, it was possible to monitor changes in the terpene profile of kaffir lime juice under different storage conditions, namely, 4 °C, 20 °C, and 35 °C. The identification of key decomposition products was achieved using gas chromatography–mass spectrometry (GC–MS) and a data mining protocol. It was followed by tracing those products in different storage conditions using a high-throughput proton transfer reaction mass spectrometry (PTR–MS) approach. Based on our findings, degradation pathways were presented, showing that the main products resulting from storage are *p*-cymene, *p*-cymenene, terpinene-4-ol, and α-terpineol. It was shown that conversion to *p*-cymenene occurs after 5 days of storage. Terpinene-4-ol and α-terpineol were found to be the final products of the conversion at all temperatures. Changes in the composition of terpenes are important from the point of view of their bioactive properties.

## 1. Introduction

The problem of postproduction residues in the production of fruit products, such as juices or jams, is an important issue. The storage of citrus waste poses a problem of landfill gas or leachate emission, the generation of which is subject to strict regulations and generates many costs [1]. Citrus fruits are a rich source of many substances, especially terpenes, which can be used for further applications. Terpenes are secondary metabolites produced by plants. Many of them have bioactive properties and are, therefore, widely used in the cosmetic industry [2]. Because of their pleasant sensory properties, they are often included in fragrance compositions. Moreover, terpenes also have bactericidal or virucidal activities [3]. The terpene profile isolated from citrus fruits depends on many aspects, mainly the degree of fruit maturation. Different plant varieties and species produce different terpenes, and the mechanisms of their production and decomposition are not similar in certain plants. Therefore, there is a need to study individual terpene transformation mechanisms for particular plants. As we mentioned in previous studies, the knowledge of the transformations that take place in fruit juices and, therefore, the changes in terpene concentrations during storage, allows for the determination of changes in the functional properties of fruit (degradation products may have different bioactive and sensory properties). This is important since their profiles in fruits vary depending on growing conditions, degree of maturity, or storage conditions [4].

Regarding citrus waste, the problem of management is particularly important in the case of kaffir lime (*Citrus hystrix*). An overview of the production, post-harvest, and marketing of kaffir lime was provided by Budiarto et al. [5], who reported that kaffir lime is a popular citrus fruit in Southeast Asia, mainly because of its leaves and outer part of the peel, which are used as spices and as an ingredient in many dishes. Despite its culinary uses, the peel is considered waste after the leaves have been picked [6]. In Thailand, it is sometimes used as a detergent for clothes and hair [7]. In Cambodia, it is common to add kaffir lime pieces to holy water during religious ceremonies. However, these are occasional applications. Kaffir lime juice is also used in folk medicine in some Asian countries [8]. The juice is not consumed because of its bitter taste, so there is no commercial production. In the case of kaffir limes, 80% of the fruit crops are industrial waste. In the literature, there are studies on the chemical composition of kaffir lime juice and its aromatic and health-promoting properties [3,4,9]. However, to the best of our knowledge, no one has ever investigated kaffir lime juice in terms of the compositional changes that occur under different storage conditions. Li et al. studied changes in aroma compounds in orange juice during storage [10], but there are no similar studies for kaffir lime juice. As kaffir lime juice can have many uses, as indicated in the abovementioned articles, this study may be crucial in the context of importing kaffir limes from the area where they are grown. Kaffir lime fruits are harvested from June to July [5]; therefore, the maximum temperature in the harvesting areas is 35 °C. The high temperature of fruit storage in non-refrigerated conditions contributes to numerous changes in the terpene profile of the fruit, resulting in changes in functional, health-promoting, and sensory properties.

So far, the decomposition/degradation products of terpenes have been studied using nuclear magnetic resonance (NMR), Fourier-transform infrared spectroscopy (FTIR), ultraviolet (UV) analysis, gas chromatography (GC) [11], and, mainly, gas chromatography–mass spectroscopy (GC–MS) [12]. In general, the degradation of terpenes occurs in four ways including (1) epoxidation; (2) oxidative cleavage of carbon–carbon double bonds; (3) allylic oxidation into alcohols, ketones, and aldehydes; and (4) dehydrogenation into aromatic systems [13]. In the context of shelf-life determination, where quick and robust methods are often required, high-throughput methods are sought. Separation in the GC column is a factor that prolongs and causes low throughput of the analysis. In recent years, the application of direct injection mass spectrometry methods in VOC analysis has been presented as a good alternative in this field [14]. One of them is proton transfer reaction mass spectrometry (PTR–MS), which is gaining popularity. Although this technique was developed almost exclusively for the detection of gaseous organic compounds in air, it has become a remarkably versatile tool with applications in many areas of science and technology. Most published papers are based on the study of VOCs in the environment, particularly for atmospheric science, but PTR-MS application in food science/technology and health science has increased in recent decades. One of the main advantages of PTR-MS is that PTR–MS fingerprints can be obtained very quickly, which significantly accelerates the speed of testing because there is no need to separate individual sample components [15]. In addition, this technique allows real-time quantification while maintaining good sensitivity (even in ppq_v_ levels), a wide dynamic range, and broad applicability (most VOCs, except, e.g., short-chain alkanes) [16,17].

In this paper, the analysis of kaffir lime juice stored under different conditions, namely, 20 °C (room temperature), 4 °C (refrigerator conditions), and 35 °C (average daily air temperature of places where kaffir lime is grown during the kaffir lime harvest) was performed. The aim of this study was to assess the decomposition/degradation products of selected terpenes in the kaffir lime juice. Therefore, the determination of the terpenes and their degradation products was performed using solid phase microextraction (SPME) and GC–MS. Data were processed using chemometrics, resulting in the selection of the major terpene degradation products. In the next step, high-throughput headspace analysis of juice was performed using PTR–MS. To the best of our knowledge, this was the first attempt to determine the terpene degradation pathway using GC–MS and PTR–MS. Providing research results in the field of food chemistry may contribute to the increasing interest in real-time food monitoring, especially using the PTR-MS technique. Because of the health-promoting effects of terpenes contained in kaffir lime juice, the obtained results create new opportunities for the potential use of kaffir lime juice extracts in the pharmaceutical industry, which will contribute to better disposal of waste after leaf picking.

## 2. Results and Discussion

### 2.1. Potential Degradation Products

Numerous studies have demonstrated the rich terpene profiles of citrus fruits [18]. The terpene profile of kaffir lime juice has been studied previously [4]. Based on the changes in the content of the selected compounds, we decided to determine the changes that occurred in the juice as a result of storage and then to determine the possible decomposition/degradation pathways and compare them with previous findings from the literature. In the first step, chemical compounds were selected based on the literature as potential products of terpene degradation (Table S1). A literature review showed that terpenes degrade into various products. Chemical compounds with molecular masses of 150.22 Da (eucarvone, thymol, carvone, verbenone, myrtenal, carvacrol), 152.23 Da (verbenol, pinocarveol, pineol, myrtenol, perillyl alcohol), 154.25 Da (α-terpineol, 1,4-cineole, eucalyptol, β-terpineol, terpinene-4-ol), and 172.26 Da (trans-1,8-p-menthanediol, cis-1.8-p-menthanediol) are formed. Conversion reactions of bicyclic monoterpenes to monocyclic monoterpenes also occur, resulting in compounds with masses of 132.20 Da (m-cymenene, *p*-cymenene), 134.21 Da (*p*-cymene, *p*-mentha-1.5.8-triene), and 136.23 Da (y-terpinene, α-terpinene, limonene, α-phellandrene, terpinolene, β-phellandrene, camphene, β-myrcene, cis-ocimene, trans-ocimene) [13].

### 2.2. SPME-GC–MS Analysis

#### 2.2.1. Determination of Terpenes and their Degradation Products Using GC–MS

The changes in the distribution of particular terpenes after storage are shown in Table 1. Under the given conditions, conversion reactions of bicyclic monoterpenes to monocyclic monoterpenes mainly take place. Under refrigerated conditions, there are no significant changes in composition, regardless of the storage time. At room temperature and 35 °C, a decrease in the β-pinene content is observed. The α-terpineol content increases with the storage time at room temperature. An increase in limonene content is observed after 7 days of storage at both room temperature and 35 °C. An increase in the content of *p*-cymene and a decrease in α-phellandrene, γ-terpinene, α-terpinene, and terpinolene is observed after 10 days of storage at both 20 °C and 35 °C. This may indicate the conversion of the α-phellandrene, γ-terpinene, α-terpinene, and terpinolene into *p*-cymene. The formation of *p*-cymenene from limonene was observed at higher storage temperatures with a greater increase at higher temperatures. Considering the results below, the potential degradation pathway of terpenes may be similar to lime essential oils, which was presented by Jakab [19].

**Figure 1:**
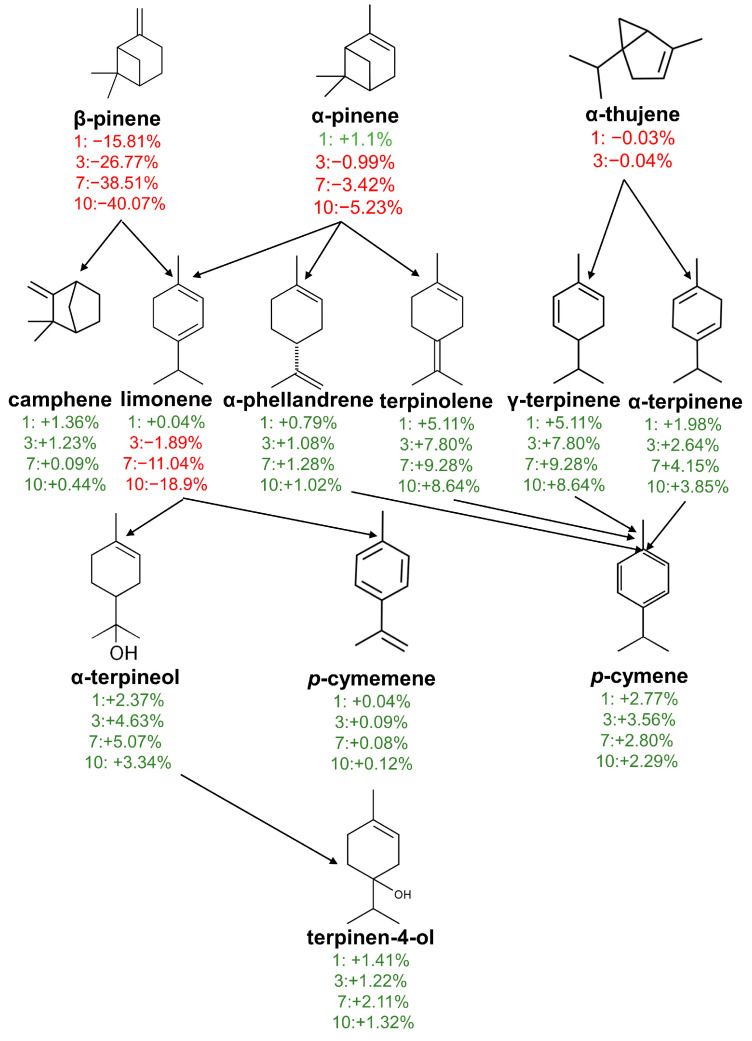
Terpene degradation pathway of kaffir lime juice during storage for 1, 3, 7, and 10 days at 35 °C. The percentage data indicate the changes in the compound yields of kaffir lime juice in relation to day 0.

The proposed degradation pathway of the main terpenes in kaffir lime juice is presented in Figure 1.

The scheme is based on results obtained at a storage temperature of 35 °C. During thermo-oxidative decomposition, bicyclic compounds (α-pinene, β-pinene, α-thujene) were decomposed into monocyclic compounds. This was most evident in the case of β-pinene, the amount of which was reduced to 0.36% after 10 days of storage at 35 °C. The above studies confirm the hypothesis that bicyclic monoterpenes are very sensitive to heat [19]. The formation of *p*-cymenene was mainly related to the dehydrogenation of limonene by opening the cyclohexadiene ring. The increase in *p*-cymene content was caused by the decomposition of terpinolene, y-terpinene, α-phellandrene, and α-terpinene.

#### 2.2.2. Statistical Analysis

Although there were some differences among each storage temperature, the chromatographic data were processed using statistical methods. Considering the results of the heterogeneity in the regression test (Table 2), significant differences (*p* < 0.05) were found among all storage temperatures determined for β-pinene, *p*-cymenene, and terpinolene. While β-pinene was present in higher amounts at 4 °C and 20 °C, *p*-cymenene and terpinolene were present in higher amounts at 20 °C and 35 °C. Other terpenes such as β-myrcene, α-phellandrene, α-terpinene, and limonene were present in higher proportions at 35 °C.

**Figure 2:**
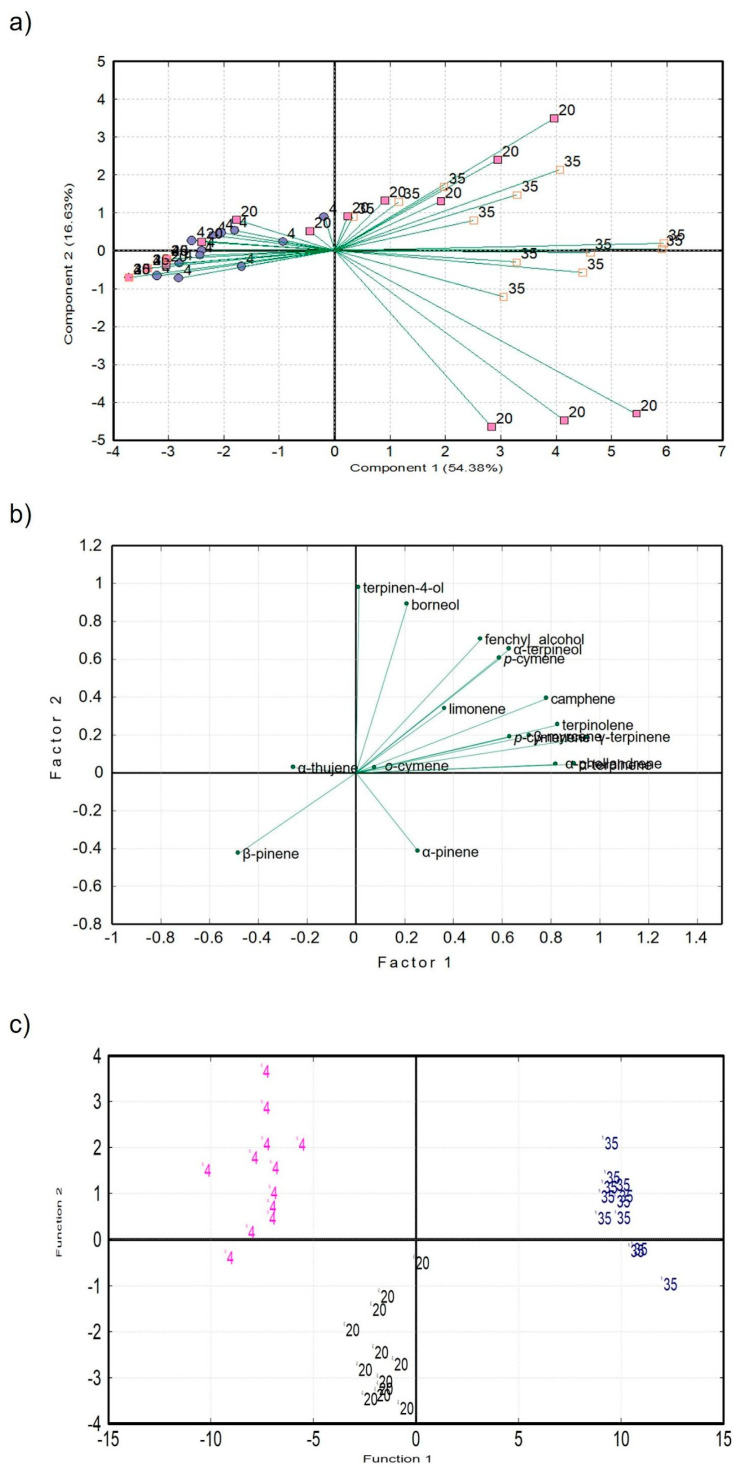
(**a**) Principal component analysis of kaffir lime juice stored at different temperatures based on the terpene profile. (**b**) Plot of factors (varimax rotation) indicating the importance of individual terpenes for kaffir lime juice discrimination. (**c**) Canonical discriminant analysis of kaffir lime juice according to the storage temperature based on the terpene profile.

Principal component analysis was performed in order to obtain an overall impression of the correlation between storage temperatures. The plot of the principal components (Figure 2a) shows partially differentiated groups of eigenvectors belonging to the different temperatures. Although the groups are not separated, the grouping tendency indicates that the identified terpenes can be used for the purpose of kaffir lime juice discrimination. The first two principal components (PCs) described more than 72% of the total variability in the dataset. The first PC was mainly represented by terpinolene, fenchyl alcohol, α-terpineol, and β-pinene, while the second PC was represented by α-pinene, terpinene-4-ol, and borneol. The importance of these terpenes was also confirmed by principal component factoring (PCF, Figure 2b) with varimax rotation, which resulted in a plot of factors corresponding to the PCA data projection (Figure 2a).

The result of canonical discriminant analysis (CDA, Figure 2c) shows the discrimination of samples into three discrete zones—the discrimination score achieved 100% accuracy in correctly classifying samples according to the storage temperature. As in the case of PCA and PCF, terpinen-4-ol, borneol, α-pinene, and α-thujene showed the highest discriminant power. On the contrary, the stepwise discriminant analysis identified *p*-cymenene, α-thujene, and α-terpineol as the most important, and the discrimination score reached more than 86% accuracy.

### 2.3. PTR–MS Analysis

#### 2.3.1. Fragmentation Pattern

Terpenes tend to fragment in the ionization region, namely, the drift tube of the PTR-MS instrument. Therefore, the determination of the optimal E/N value (where E is the electric field strength and N is the numerical density of the buffer gas in the drift chamber) is an important part of studying terpene compounds. There are reports on the fragmentation patterns of selected terpenes determined by the PTR–MS technique [20,21]. However, the transmission coefficients and thus the ion product distributions in PTR–MS depend on the type of mass spectrometer, so fragmentation patterns should be determined for each instrument [22]. Based on the library of spectra prepared, it was found that in the case of this group of compounds, their differentiation and determination by the PTR–MS technique may be impossible because of too similar fragmentation patterns. In any case, ions 81 and 137 are the dominant fragment ions, which is in agreement with previous literature reports [23]. For other terpenes present in the sample, it can be assumed that their fragmentation patterns will be similar.

The fragmentation patterns of many terpenes have been determined previously [21,23]. Based on the above results, we decided to determine the changes in the total content of compounds of *m*/*z* 137 and 81 (characteristic for terpenes) and 150.22 Da, 152.23 Da, 154.25 Da, and 172.26 Da (characteristic for terpene degradation products) during the storage of fruit under different conditions and at different times. Based on the chromatographic measurements, the identification of the detected chemical compounds was performed and the percentage content of selected terpenes was determined in order to predict their metabolic pathway. The generated spectral library was used to select the best E/N value to measure the terpene group. The highest relative contents of the main fragmentation ions (81 and 137) were recorded at E/N equal to 120 (Table S2 in Supplementary Materials); therefore, this value was used in further analyses. This is in agreement with current literature reports [21,23]. Higher E/N values can lead to greater fragmentation, while lower values can lead to increased hydronium/water clustering, which in turn makes data difficult to interpret [24].

#### 2.3.2. High-Throughput Headspace Analysis of Kaffir Lime Juice Using PTR–MS

Dynamic headspace analysis using PTR–MS was performed to develop a high-throughput and convenient method for the assessment of terpene conversion in kaffir lime juice. The justification for the application of the PTR-MS-based method of the proposed method is (1) direct qualitative determination of multiple VOCs, (2) short analysis—in seconds, (3) limited sample preparation—headspace analysis, and (4) analytical greenness—solvent-free approach. Moreover, many terpene degradation products are structural isomers; thus, PTR-MS measurement simplifies the interpretation and depicts the total load of all products with the same chemical structure. However, problems related to the rich headspace composition of kaffir lime juice and the behavior of terpenes in the PTR–MS drift tube had to be overcome. Terpenes tend to fragment in the drift tube along with the clustering with the H_5_O_2_^+^ (37.03 *m*/*z*) ion [20,21]. This phenomenon is shown in Table S2 (Supplementary Material), where fragment ions of 67.06, 81.07, and 95.09 *m*/*z* were registered, as well as the ion 155.14 *m*/*z*, which is the clustering product. At the same time, the ion 155.14 may represent terpinen-4-ol or α-terpineol (both C_10_H_18_O), which have been shown to be the final terpene conversion products of terpenes (C_10_H_16_). For PTR–MS analysis, ions 133.10, 135.12, and 155.14 *m*/*z* were selected as the representatives of terpene conversion products, where 133.10 *m*/*z* may represent *p*-cymenene (C_10_H_12_), and 135.12 *m*/*z* may be a product of the protonation of *p*-cymene (C_10_H_14_).

**Figure 3:**
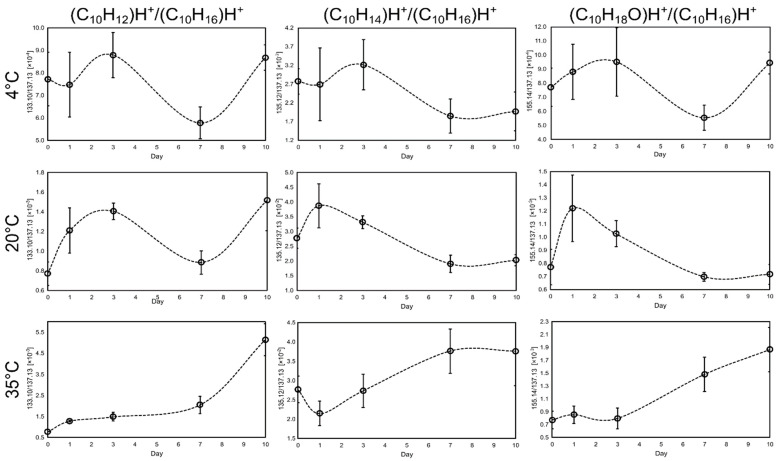
Changes in the ratio (C_10_H_12_)H^+^/(C_10_H_16_)H^+^, (C_10_H_14_)H^+^/(C10H16)H^+^, and (C_10_H_18_O)H+/(C1_0_H_16_)H^+^ ions emitted by the kaffir lime juice after storage for 10 days at three different temperatures, namely, 4 °C, 20 °C, and 35 °C. Error bars represent standard deviation; n = 5.

The ratios of the conversion product ion and terpene ion (137.13 *m*/*z*) in different storage conditions are shown in Figure 3. The rationale for describing the conversion products as a ratio is that it overcomes several problems and simplifies measurement and analysis. First, the ratio on day 0 shows a representation of the compounds in the fresh juice, and any change in the ratio indicates chemical transformations that occur during storage. It can represent both the decomposition of C_10_H_12_, C_10_H_14_, and C_10_H_18_O compounds (the ratio decreases) and the conversion of terpenes into these compounds. Second, the change in the 155.14 *m*/*z* signal may result in terpene concentration, as this could be the clustering product of terpenes. However, using the ratio value between these ions overcomes this problem, and whenever the ratio arises, the headspace concentration of the C_10_H_18_O conversion products also occurs. Finally, there is no need to measure the concentration of these volatiles or use corrected counts per second (cps) because the ratio is dimensionless. This simplifies data processing.

As can be seen in Figure 3, the ratio of different ions changes with storage time. However, an increase in the ratio with time was observed only for the storage at 35 °C. This was confirmed by GC–MS analysis, where C_10_H_12_, C_10_H_14_, and C_10_H_18_O were found to be good shelf-life indicators. At the same time, C_10_H_12_ seemed to be the best indicator since the ratio increase is the most pronounced and the standard deviations between repetitions are small.

To determine when the conversion of terpenes to *p*-cymenene occurs, the polynomial function was fitted to the obtained data (see Figure S1 in Supplementary Material). The second derivative of this function was then used to find the inflexion point. According to the obtained calculations, the conversion of terpenes to *p*-cymenene takes place after 5 days of storage (5.33 days).

## 3. Materials and Methods

### 3.1. Chemicals

Analytical terpene standards α-thujene, α-pinene, camphene, β-pinene, β-myrcene, α-phellandrene, α-terpinene, limonene, *p*-cymene, γ-terpinene, terpinolene, borneol, terpinen-4-ol, α-terpineol, and methanol (Sigma-Aldrich, St. Louis, MO, USA) were used to perform a tentative identification of the detected compounds.

### 3.2. Fruit Samples

**Figure 4:**
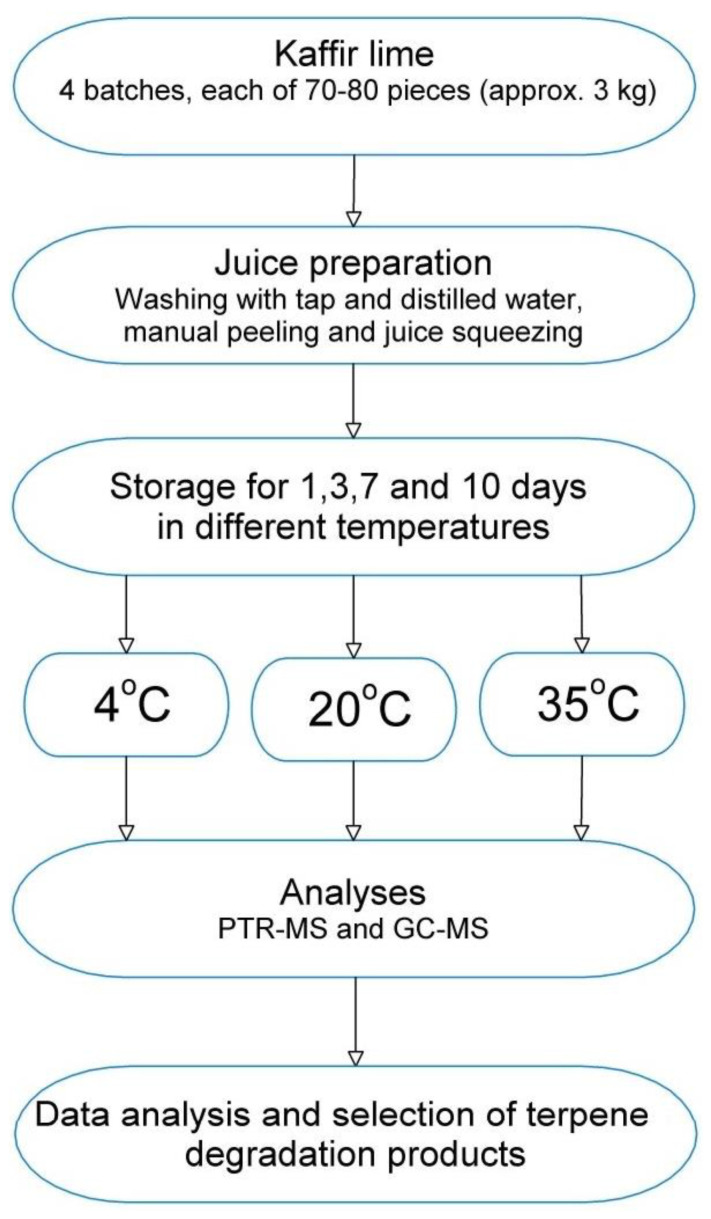
Schematic of the analytical procedure.

Kaffir lime originated from Southeast Asia. Fruits for the analysis were purchased at the local Gdańsk distribution points where they were imported from Thailand. Before the analysis, the fruits were stored at 4 °C. The fruits were washed under running water and then rinsed with distilled water. The peel was manually separated from the pulp. The juice was manually squeezed and filtered through filter paper (75 g/m^2^). Samples of 5 g of kaffir lime juice were placed in 20 mL glass headspace vials and sealed with a PTFE–silicone membrane. The procedure used during the experiment is shown in Figure 4.

### 3.3. SPME-GC Analysis

#### 3.3.1. SPME Extraction

The headspace solid phase microextraction (HS-SPME) technique was used for the isolation and enrichment of analytes. The sample was incubated at 45 °C for 5 min prior to extraction. The SPME extraction of terpenes was performed at 45 °C for 30 min with constant agitation using a DVB/CAR/PDMS (divinylbenzene/carboxen/polydimethylsiloxane) coated fiber—50/30 μm thickness, 2 cm length (Sigma-Aldrich, St. Louis, MO, USA)—followed by thermal desorption of the analytes at the temperature of 250 °C for 5 min in the GC injector. Between each analysis, the fiber was desorbed at 250 °C for 5 min. A MPS autosampler (Gerstel Co., Mülheim, Germany) was used for the extraction step.

#### 3.3.2. GC–MS

Chromatographic analysis was performed according to a previously established procedure [10]. Briefly, the analysis was performed using an Agilent 7980 gas chromatograph (Agilent Technologies, Palo Alto, CA, USA) coupled to a Pegasus 4D time-of-flight mass spectrometer (LECO Corp., St. Joseph, MI, USA). The separation was achieved using the following oven temperature program: initial temperature 60 °C, ramped at 7.5 °C/min to 150 °C, then 15 °C/min to 250 °C, and held for 2 min. The total time of analysis was 18 min. The back inlet temperature was 200 °C, and the transfer line and ion source temperatures were set at 250 °C. The injector was operated in splitless mode. Helium (N6.0 class) was used as the carrier gas at a flow rate of 1.0 mL/min. The detector voltage was 1716 V with an ionization energy of 70 eV. Mass spectra were collected from *m*/*z* 35 to 500 at 10 Hz. The acquisition delay was 300 s. The compounds detected were confirmed by analytical standards, both by retention time and by comparison of mass spectra. Chemical structures, such as *o*-cymene, *p*-cymene, *p*-cymemene, and fenchyl alcohol were tentatively identified by comparison with hits from MS spectra libraries (NIST 11 and Wiley).

### 3.4. PTR–MS Analysis

The high-throughput headspace analysis of kaffir lime juice was performed using the PTR-TOF1000ultra instrument (Ionicon GmbH, Innsbruck, Austria). Different E/N values ranging from 80 to 180 Td were tested to find the optimal value for further analysis. The optimal E/N was found to be 120 Td. The fragmentation pattern was performed using 100 μg/mL single compound aqueous solution of terpenes, namely, citrolellal, limonene, terpinen-4-ol, β-pinene, α-terpinene, α-terpineol, α-pinene, y-terpinene, citral, and linalool. Approximately 50 sccm (cm^3^ min^−1^ at standard temperature, 273 K, and pressure, 1 atm) of the filtrated air was transferred in a dynamic mode through the 20 mL headspace vial containing 5 g of sample, and the headspace was transferred to the PTR–MS instrument. Filtrated air was obtained using Supelpure^®^ HC Hydrocarbon Trap (Sigma-Aldrich Co.). The PTR–MS transfer line was heated to 70 °C. The total analysis time for one sample was approx. 1 min, with an acquisition speed of 1 spectra/s. Spectra were recorded using IoniTOF v3.0 (Ionicon GmbH, Innsbruck, Austria), and the data were processed in PTR–MS Viewer v.3.4.2.1 (Ionicon GmbH, Innsbruck, Austria).

### 3.5. Statistical Analysis

Experimental data were processed using the Unistat^®^ v.6.0 statistical package (Unistat, London, United Kingdom). The analysis of variance (ANOVA) heterogeneity in regression test by multiple comparisons for intercepts (95% Tukey HSD interval) was used to assess statistical significance among storage temperatures. Intercepts were considered significantly different at *p* < 0.05. From the multidimensional pattern recognition techniques, the principal component analysis (PCA), principal component factoring (PCF), stepwise discriminant analysis, and canonical discriminant analysis (CDA) were used to interpret and visualize the differences among the storage temperatures and to define the most appropriate volatiles for the discrimination of kaffir lime juice. The convergence criteria of the discriminant analysis were chosen for a standardized proximity matrix with a maximum number of iterations. The following selection criteria of stepwise discriminant analysis were used: tolerance—0.001 and F statistic: F to enter—3.8416, F to remove—2.7056.

MS Excel was used for ion signal correction and PTR–MS data processing.

## 4. Conclusions

Based on the presented approach, it was possible to determine the potential decomposition pathways of selected terpenes in kaffir lime samples stored at different temperatures. *p*-cymene, *p*-cymenene, terpinene-4-ol, and α-terpineol were selected as the degradation products of the primary terpenes of kaffir lime juice. The developed pathways also complement the current knowledge on the transformation of terpenes in citrus fruit juices. This research may contribute to the increase in the popularity of kaffir lime and consequently reduce the waste generated after harvesting leaves and using only the peels for culinary purposes. Moreover, the high-throughput PTR-MS-based approach was efficient in the determination of the time needed for terpene conversion. Such an approach can be used to estimate the shelf-life of the kaffir lime juice accurately and in the processing and manufacturing of kaffir lime-based products.

## Figures and Tables

**Table 1:** Changes in terpene distribution (% of total peak area) during the storage of kaffir lime juice under different conditions.

Chemical Compound	ID	Total Peak Area (%)												
			4 °C				20 °C				35 °C			
		Control	Day 1	Day 3	Day 7	Day 10	Day 1	Day 3	Day 7	Day 10	Day 1	Day 3	Day 7	Day 10
α-thujene	RT, MS	0.12122 ± 0.013	0.136 ± 0.033	0.137 ± 0.022	0.113 ± 0.020	0.126 ± 0.017	0.283 ± 0.036	0.065 ± 0.068	0.095 ± 0.013	0.0978 ± 0.0011	0.0884 ± 0.0042	0.08 ± 0.02	nd.	nd.
α-pinene	RT, MS	8.20 ± 0.48	7.61 ± 0.73	8.54 ± 0.81	7.4 ± 1.5	8.8 ± 2.1	8.0 ± 1.0	7.8 ± 1.0	12.6 ± 2.0	2.30 ± 0.55	10.10 ± 0.52	7.21 ± 1.15	4.78 ± 0.31	2.97 ± 0.49
camphene	RT, MS	1.175 ± 0.069	1.12 ± 0.23	1.34 ± 0.04	1.75 ± 0.37	1.56 ± 0.18	1.18 ± 0.25	1.81 ± 0.30	3.29 ± 0.60	2.26 ± 0.51	2.53 ± 0.10	2.40 ± 0.44	2.07 ± 0.40	1.61 ± 0.32
β-pinene	RT, MS	41.3 ± 1.4	42.53 ± 4.0	42.7 ± 1.9	40.6 ± 3.2	43.3 ± 1.1	42.8 ± 2.7	29.1 ± 3.6	20.2 ± 5.1	1.15 ± 0.87	25.5 ± 1.5	14.56 ± 0.62	2.82 ± 0.48	0.362 ± 0.072
β-myrcene	RT, MS	nd.	nd.	nd.	nd.	nd.	nd.	0.530 ± 0.019	3.01 ± 0.16	1.95 ± 0.22	3.04 ± 0.21	3.16 ± 0.31	2.80 ± 0.24	4.08 ± 0.99
α-phellandrene	RT, MS	0.81 ± 0.10	0.89 ± 0.11	1.335 ± 0.033	1.41 ± 0.27	1.22 ± 0.17	1.40 ± 0.18	1.57 ± 0.14	2.09 ± 0.46	1.38 ± 0.11	1.604 ± 0.085	1.89 ± 0.17	2.09 ± 0.12	1.83 ± 0.29
α-terpinene	RT, MS	0.68 ± 0.15	2.53 ± 0.41	1.89 ± 0.19	1.59 ± 0.27	2.49 ± 0.40	3.55 ± 0.71	2.61 ± 0.65	3.21 ± 0.44	1.79 ± 0.15	2.66 ± 0.40	3.32 ± 0.50	4.83 ± 0.29	4.53 ± 0.29
*o*-cymene	MS	1.90 ± 0.15	1.80 ± 0.13	1.74 ± 0.10	1.61 ± 0.27	0.97 ± 0.24	0.82 ± 0.09	2.35 ± 0.17	1.60 ± 0.25	1.70 ± 0.28	1.93 ± 0.30	2.88 ± 0.09	1.33 ± 0.10	1.482 ± 0.094
limonene	RT, MS	17.9 ± 2.4	13.9 ± 3.3	12.7 ± 2.6	21.9 ± 3.5	16.66 ± 1.7	13.1 ± 1.9	13.1 ± 2.1	23.32 ± 3.2	28.5 ± 3.2	17.9 ± 1.6	16.0 ± 1.6	28.94 ± 5.77	36.8 ± 6.2
*p*-cymene	MS	nd.	2.23 ± 0.34	2.61 ± 0.13	2.64 ± 0.86	1.79 ± 0.05	2.19 ± 0.29	2.77 ± 0.46	2.61 ± 0.29	4.14 ± 0.41	2.77 ± 0.13	3.56 ± 0.47	2.80 ± 0.55	2.285 ± 0.086
γ-terpinene	RT, MS	13.9 ± 1.3	13.24 ± 0.70	15.22 ± 0.65	15.71 ± 0.90	12.51 ± 0.49	15.8 ± 2.0	17.01 ± 0.71	19.8 ± 1.9	14.4 ± 2.0	17.0 ± 2.2	19.4 ± 1.9	19.4 ± 2.0	18.64 ± 3.1
*p*-cymenene	MS	nd.	nd.	nd.	nd.	nd.	nd.	0.0508 ± 0.0016	0.045 ± 0.002	0.052 ± 0.021	0.0432 ± 0.0043	0.09 ± 0.0025	0.0785 ± 0.0055	0.121 ± 0.012
terpinolene	RT, MS	2.49 ± 0.20	2.36 ± 0.45	4.0 ± 1.1	4.39 ± 0.76	3.63 ± 0.24	3.99 ± 0.42	6.88 ± 0.29	10.09 ± 0.55	7.4 ± 1.7	7.60 ± 0.88	10.3 ± 1.1	11.8 ± 1.2	11.1 ± 1.6
fenchyl alcohol	MS	0.32 ± 0.20	0.31 ± 0.05	0.401 ± 0.091	0.490 ± 0.077	0.435 ± 0.066	0.361 ± 0.075	0.64 ± 0.12	1.021 ± 0.072	1.65 ± 0.39	0.72 ± 0.16	1.018 ± 0.087	1.30 ± 0.12	1.11 ± 0.066
borneol	RT, MS	0.28 ± 0.20	0.301 ± 0.042	0.282 ± 0.043	0.349 ± 0.039	0.311 ± 0.066	0.229 ± 0.034	0.437 ± 0.073	0.665 ± 0.045	1.72 ± 0.15	0.535 ± 0.057	0.647 ± 0.037	0.816 ± 0.041	0.732 ± 0.051
terpinen-4-ol	RT, MS	4.72 ± 0.83	5.00 ± 0.64	5.01 ± 1.0	5.20 ± 0.61	4.57 ± 0.44	6.8 ± 1.1	4.81 ± 0.77	5.75 ± 0.71	17.5 ± 2.3	6.1 ± 1.0	5.9 ± 1.0	6.8 ± 1.4	6.0 ± 1.2
α-terpineol	RT, MS	1.85 ± 0.15	2.280 ± 0.052	2.44 ± 0.26	2.40 ± 0.25	2.47 ± 0.38	2.52 ± 0.48	4.52 ± 0.66	6.55 ± 0.97	8.2 ± 1.5	4.58 ± 0.65	6.48 ± 0.26	6.9 ± 1.6	5.19 ± 0.67

ID—compounds identification, MS—identification based on comparing of compound’s mass spectra with mass spectra from the library, RT—identification based on comparing the compounds’ retention time with standards’ retention time, nd.—not detected.

**Table 2:** Statistically significant differences among the storage temperatures of kaffir lime juice evaluated by the ANOVA heterogeneity in regression test by multiple comparisons for intercepts (*p* < 0.05).

Parameter	Comparison ^a^	Difference	Standard Error	Probability
α-thujene	20–35	0.0760	0.0114	0.0001
	4–35	0.0700	0.0101	0.0002
camphene	35–4	0.5680	0.1307	0.0102
	20–4	0.5540	0.1348	0.0124
β-pinene	4–35	25.1560	0.1566	0.0000
	20–35	11.0080	0.1564	0.0000
	4–20	14.1480	0.1559	0.0000
β-myrcene	35–4	2.0780	0.2460	0.0000
	35–20	1.5180	0.2459	0.0002
α-phellandrene	35–4	0.5100	0.0889	0.0006
	35–20	0.1940	0.0889	0.0413
α-terpinene	35–4	1.3680	0.2317	0.0004
	35–20	0.8360	0.2329	0.0377
limonene	35–4	6.9000	1.0472	0.0001
	35–20	4.3500	1.0468	0.0145
γ-terpinene	35–4	3.5340	0.5663	0.0002
	20–4	2.0680	0.5598	0.0350
*p*-cymenene	35–4	0.0660	0.0040	0.0000
	20–4	0.0300	0.0044	0.0000
	35–20	0.0360	0.0038	0.0000
terpinolene	35–4	5.2880	0.4622	0.0000
	20–4	2.7940	0.4578	0.0003
	35–20	2.4940	0.4601	0.0013
fenchyl alcohol	35–4	0.5020	0.0445	0.0000
	20–4	0.4060	0.0436	0.0000
borneol	20–4	0.3620	0.0405	0.0000
	35–4	0.2980	0.0387	0.0000
terpinen-4-ol	20–4	3.0060	0.5401	0.0009
	20–35	1.9800	0.5412	0.0342

^a^ The first value in the comparison represents significantly higher mean values, e.g., 20–35, which means that the mean values of α-thujene were higher at the storage temperature of 20 °C.

## Data Availability

The data presented in this study are available upon request from the corresponding author. The data are not publicly available because of privacy reasons.

## Supplementary Materials

The following supporting information can be downloaded at https://www.mdpi.com/article/10.3390/molecules29133241/s1, Figure S1: Diagram of a polynomial function with the second derivatives of the data obtained after PTR–MS analysis; Table S1: Potential degradation products of terpenes under different storage conditions; Table S2: Relative content of fragmentation ions of selected terpenes determined using the PTR–MS technique. (E/N = 120) normalized for the 137.13 ion. References [11,12,13,19,25,26,27,28,29,30,31] are cited in the Supplementary Materials.
