# Supplementary material for: Evaluation of Terpene Decomposition in Kaffir Lime Juice during Storage Using Gas Chromatography–Mass Spectrometry and Proton Transfer Reaction–Mass Spectrometry
Source: Molecules. 2024 Jul 8;29(13):3241. doi: 10.3390/molecules29133241 (PMC11243363; doi:10.3390/molecules29133241)
Supplement: Supplementary file 1 [file molecules-29-03241-s001.zip › molecules-3050559-supplementary.pdf]

Supplementary Materials

Evaluation of Terpene Decomposition in Kaffir Lime Juice During Storage Using GC–MS and PTR–MS

Martyna Lubinska-Szczygeł [1,*], Żaneta Polkowska [1,*], Blanka Tobolkova [2], Tomasz Majchrzak [1], Martin Polovka [2], Parichart Promchote [3] and Shela Gorinstein [4]

[1] Gdańsk University of Technology, Faculty of Chemistry, Department of Analytical Chemistry, Gdańsk 80-233, Poland;

[2] Department of Chemistry and Food Analysis, National Agricultural and Food Centre-Food Research Institute, 824 75 Bratislava, Slovakia

[3] Department of Agronomy, Faculty of Agriculture, Kasetsart University, Chatuchak, Bangkok, 10900, Thailand

[4] Institute for Drug Research, School of Pharmacy, Faculty of Medicine, The Hebrew University of Jerusalem, Jerusalem 9112001, Israel; shela.gorin@mail.huji.ac.il (S.G.) *

*Correspondence: martyna.lubinska@pg.edu.pl (M.L-Sz.); Zaneta.polkowska@pg.edu.pl (Ż.P.)

Table S1. Potential degradation products of terpenes under different storage conditions

Primary terpene	Potential degradation product	Chemical formula	Molecular mass (Da)	Lit.
α-thujene	y-tepinene	$C_{10}H_{16}$	136.23	[19]
	α-terpinene	$C_{10}H_{16}$	136.23	[19]
α-pinene	verbenol	$C_{10}H_{16}O$	152.23	[11]
	pinocarveol	$C_{10}H_{16}O$	152.23	[11]
	pinenol	$C_{10}H_{16}O$	152.23	[11]
	myrtenol	$C_{10}H_{16}O$	152.23	[11]
	verbenone	$C_{10}H_{14}O$	150.22	[11]
	pinocarvone	$C_{10}H_{14}O$	150.22	[11]
	α-pinene oxide	$C_{10}H_{14}O$	150.22	[11]
	myrtenal	$C_{10}H_{14}O$	150.22	[11]
	limonene	$C_{10}H_{16}$	136.23	[25]
	α-phellandrene	$C_{10}H_{16}$	136.23	[25]
	terpinolene	$C_{10}H_{16}$	136.23	[25]
	β-phellandrene	$C_{10}H_{16}$	136.23	[25]
camphene	verbenone	$C_{10}H_{14}O$	150.22	[13]
	camphenilone	$C_9H_{14}O$	138.21	[13]
	o-cymene	$C_{10}H_{14}$	134.21	[13]
	cumene	C_9H_{12}	120.19	[13]
β-pinene	β-phellandrene	$C_{10}H_{16}$	136.23	[19]
	limonene	$C_{10}H_{16}$	136.23	[19]
	p-cymene	$C_{10}H_{14}$	134.21	[19]
	camphene	$C_{10}H_{16}$	136.23	[19]
β-myrcene	α-terpineol	$C_{10}H_{18}O$	154.25	[12]
α-phellandrene	*p*-mentha-1.5-dien-7-oic acid	$C_{10}H_{14}O$	166.23	[26]
	p-cymen-7-oic acid	$C_{10}H_{12}O_2$	164.08	[26]

α-terpinene	*p*-cymene	$C_{10}H_{14}$	134.21	[13]
	thymol	$C_{10}H_{14}O$	150.22	[13]
	carvacrol	$C_{10}H_{14}O$	150.22	[13]
	3-(1-methylethyl)-6-oxo-2-heptanal	$C_{10}H_{16}O_2$	168.23	[13]
	3,7-dimethyl-6-oxo-2-octenal	$C_{10}H_{16}O_2$	168.23	[13]
	1,4-cineole	$C_{10}H_{18}O$	154.25	[13]
	eucalyptol	$C_{10}H_{18}O$	154.25	[13]
limonene	*p*-cymenene	$C_{10}H_{12}$	132.20	[13]
	p-cymene	$C_{10}H_{14}$	134.21	[13]
	p-mentha1.5.8-triene	$C_{10}H_{14}$	134.21	[13]
	eucarvone	$C_{10}H_{14}O$	150.22	[13]
	thymol	$C_{10}H_{14}O$	150.22	[13]
	p-mentha-1-(7).8-dien-2-ol	$C_{10}H_{16}O$	152.23	[13]
	perillyl alcohol	$C_{10}H_{16}O$	152.23	[13]
	carvone	$C_{10}H_{14}O$	150.22	[13]
	α-terpineol	$C_{10}H_{14}O$	154.25	[27]
y-terpinene	*p*-cymene	$C_{10}H_{14}$	134.21	[25]
	p-menthan-1.4-dien-9-ol	$C_{10}H_{16}O$	152.23	[25]
	p-cymen-9-ol	$C_{10}H_{14}O$	150.22	[25]
	p-penthan-1,4-dien-7-oic acid	$C_{10}H_{14}O$	166.23	[25]
	p-cymen-7-oic acid	$C_{10}H_{12}O_2$	164.08	[25]
	p-menthene-1.2.-diol	$C_{10}H_{20}O_2$	172.26	[25]
terpinolene	*m*-cymenene	$C_{10}H_{12}$	132.20	[28]
linalool	β-myrcene	$C_{10}H_{16}$	136.23	[29]
	limonene	$C_{10}H_{16}$	136.23	[29]
	terpinolene	$C_{10}H_{16}$	136.23	[29]
	α-terpinene	$C_{10}H_{16}$	136.23	[29]
	cis-ocimene	$C_{10}H_{16}$	136.23	[29]
	trans-ocimene	$C_{10}H_{16}$	136.23	[29]
	α-terpineol	$C_{10}H_{14}O$	154.25	[27]
terpinene-4-ol	1,2,4-trihydroxymenthane	$C_{10}H_{20}O_3$	188.26	[30]
	p-cymene	$C_{10}H_{14}$	134.21	[30]
α-terpineol	*trans*-1,8-*p*-menthanediol	$C_{10}H_{20}O_2$	172.26	[31]
	cis-1,8-*p*-menthanediol	$C_{10}H_{20}O_2$	172.26	[31]
	eucalyptol	$C_{10}H_{14}O$	154.25	[31]
	8-hydroxy-8,9-dihydro-carvone	$C_{10}H_{16}O_2$	168.23	[31]
	β-terpineol	$C_{10}H_{14}O$	154.25	[31]
	terpinen-4-ol	$C_{10}H_{14}O$	154.25	[31]
	8-*p*-cymenol	$C_{10}H_{14}O$	150.22	[31]

Table S2. The relative content of fragmentation ions of selected terpenes determined using the PTR-MS technique. (E / N = 120) normalized for 137.13 ion

	**Fragmentation ion m/z**				
**Chemical compound**	**67.06 ($C_5H_7^+$)**	**81.07 ($C_6H_9^+$)**	**95.09 ($C_7H_{11}^+$)**	**137.13 ($C_{10}H_{17}^+$)**	**151.11 ($C_{10}H_{15}O^+$)**
Citrolellal	3.12	107.17	29.39	100.00	23.82
Limonene	0.84	86.62	9.19	100.00	4.33
Terpinen-4-ol	3.09	99.36	25.66	100.00	2.86
β-pinene	2.96	91.32	12.23	100.00	11.11

α-terpinene	2.39	88.76	9.98	100.00	5.41
α-terpineol	2.86	103.89	26.61	100.00	9.84
α-pinene	0.58	80.05	6.38	100.00	2.11
y-terpinene	1.58	93.49	8.48	100.00	3.93
Citral	1.22	85.33	7.58	100.00	5.87
Linalool	6.90	112.46	35.13	100.00	28.29

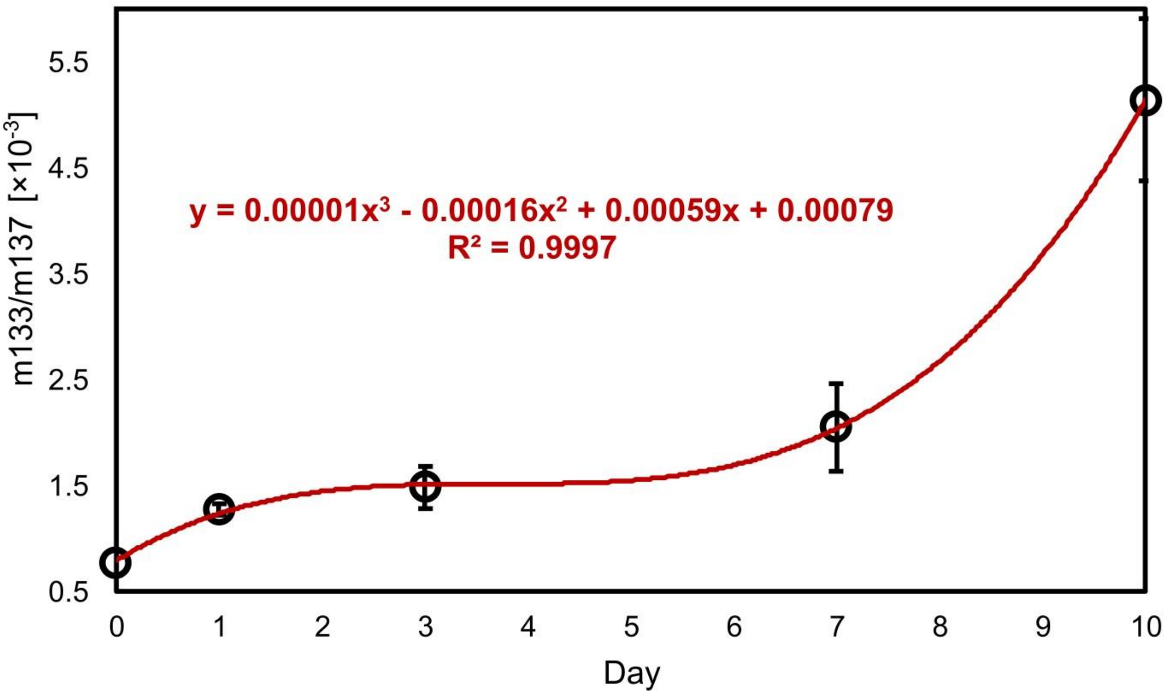

Figure S1 Diagram of a polynomial function with the second derivatives of the data obtained after PTR-MS analysis
